# Pre-Partum Diet of Adult Female Bearded Seals in Years of Contrasting Ice Conditions

**DOI:** 10.1371/journal.pone.0038307

**Published:** 2012-05-31

**Authors:** Mark A. Hindell, Christian Lydersen, Haakon Hop, Kit M. Kovacs

**Affiliations:** 1 Institute for Marine and Antarctic Studies, University of Tasmania, Sandy Bay, Tasmania, Australia; 2 Norwegian Polar Institute, Tromsø, Norway; Texas A&M University-Corpus Christi, United States of America

## Abstract

Changing patterns of sea-ice distribution and extent have measurable effects on polar marine systems. Beyond the obvious impacts of key-habitat loss, it is unclear how such changes will influence ice-associated marine mammals in part because of the logistical difficulties of studying foraging behaviour or other aspects of the ecology of large, mobile animals at sea during the polar winter. This study investigated the diet of pregnant bearded seals (*Erignathus barbatus*) during three spring breeding periods (2005, 2006 and 2007) with markedly contrasting ice conditions in Svalbard using stable isotopes (δ^13^C and δ^15^N) measured in whiskers collected from their newborn pups. The δ^15^N values in the whiskers of individual seals ranged from 11.95 to 17.45 ‰, spanning almost 2 full trophic levels. Some seals were clearly dietary specialists, despite the species being characterised overall as a generalist predator. This may buffer bearded seal populations from the changes in prey distributions lower in the marine food web which seems to accompany continued changes in temperature and ice cover. Comparisons with isotopic signatures of known prey, suggested that benthic gastropods and decapods were the most common prey. Bayesian isotopic mixing models indicated that diet varied considerably among years. In the year with most fast-ice (2005), the seals had the greatest proportion of pelagic fish and lowest benthic invertebrate content, and during the year with the least ice (2006), the seals ate more benthic invertebrates and less pelagic fish. This suggests that the seals fed further offshore in years with greater ice cover, but moved in to the fjords when ice-cover was minimal, giving them access to different types of prey. Long-term trends of sea ice decline, earlier ice melt, and increased water temperatures in the Arctic are likely to have ecosystem-wide effects, including impacts on the forage bases of pagophilic seals.

## Introduction

Sea-ice is an important habitat for many polar species. For ice-associated seals, sea ice provides a physical substrate for birthing, nursing, resting and moulting and a staging ground for mating as well as being an important habitat for prey [1], [2], [3], [4], [5]. With the demonstrated changes over recent decades in sea ice distribution and extent in the Arctic [6], [7], [8], there is increasing evidence that changes are already taking place in Arctic food webs [9] and hence in the diet, and reproductive performance of higher predators such as seals and seabirds [10], [11], [12].

Bearded seals, *Erignathus barbatus*, are a species with close ties to sea ice [13], [14], [15]. This species gives birth on small, first-year ice floes in coastal or shallow Arctic shelf areas [16] and lives in association with sea ice throughout the remainde of the year. Thus, bearded seals are likely to experience a lack of suitable ice floes (largely generated by the break-up of land-fast sea ice in the summer), which will result in a reduction of pupping and nursing habitat. Bearded seals are described as generalist, benthic foragers that eat a variety of fish and invertebrate prey, including benthic polychaetes, crustaceans, molluscs and a variety of fish [11], [17], [18] capturing prey on the bottom, or extracting it from the substrate using powerful suction and water-jetting along with their sensitive vibrissae [19], [20], [21]. Reduced sea ice cover in the Arctic is likely to affect bearded seals via changes in the dynamics and productivity of benthic communities. Less sympagic flora and fauna are likely to result in a reduction in the sedimentation of organic matter to the benthos [22], [23], [24], [25], [26] which might reduce the availability of food for bearded seals.

Relatively little is known about bearded seal diet during the crucial period of gestation, or how changing ice conditions will influence their diet. Individual seals in other species have specialist foraging strategies [27], [28], and similarly for bearded seals, there is evidence that some individuals employ benthic strategies while others eat more pelagic prey [21]. The degree of individual dietary specialisation is an important characteristic of a population [29], particularly for understanding how populations may respond to changing environmental circumstances. However, this can be difficult to quantify as it requires data on the foraging history of individual animals.

Kongsfjorden/Krossfjorden (∼79.0°N, 11.30°E) in Svalbard has historically been bearded seal breeding habitat. However, the region has demonstrated pronounced inter-annual variation in the extent of coastal sea-ice during the last 10 years. In particular, 2006 and 2007 were characterised by a lack of winter ice formation, due to a clockwise eddy overturning across the West Spitsbergen shelf edge front, leading to an up-welling of Atlantic Water onto the shelf and extensive cross-shelf exchange with the open fjords on West Spitsbergen, resulting in reduced sea-ice on the shelf [30], [31], [32]. This period thus provided a unique opportunity for a natural experiment to examine the effects of sea ice coverage on the foraging ecology of bearded seals.

The goal of this study was to quantify the diet of gestating female bearded seals over three years with widely different ice conditions. We used stable isotopes in the whiskers (vibrissae) of pups to investigate the diet of their mothers, as this offers a number of advantages over conventional stomach content analysis [33]. Adult females are very difficult to access during the late winter/ early spring months, making sampling of stomach contents and tissues which can contain dietary signals, such as blubber, blood and whiskers, problematic. Their pups on the other hand are more easily catchable both on ice floes and in the water [34]. Stable isotopes are a convenient vehicle for this study, as isotopic signatures of prey items are reflected in the tissues of predators for days to months after consumption [35]. Furthermore, if the degree of fractionation at each stage of the food web is known, broad-scale dietary reconstruction is possible [36]. Whiskers of pups growing *in utero* can provide information on the diet of mothers throughout gestation.

The specific aims of the study were to (i) describe the diets of *pre-partum* female bearded seals using stable isotopes incorporated into the whiskers of their pups during gestation, (ii) quantify the degree of individual dietary specialisation and trophic niche width of *pre-partum* females, and (iii) compare the diets among three years of contrasting ice conditions.

## Materials and Methods

The study was conducted in the Kongsfjorden/Krossfjorden region of Svalbard during the 2005, 2006 and 2007 bearded seal breeding seasons (from late April to late May in each year). In 2005, Kongsfjorden had extensive fast-ice cover (that was normal for the region throughout recorded history until the following year), which began to break-up in the first week of May, providing ample pupping habitat for the female seals. In 2006, sea ice did not form in the region, and Kongsfjorden remained open water throughout the winter, which gave gestating females feeding access to regions of the fjord normally covered by fast-ice. No sea ice floes strong enough to support an adult female and pup were available during the pupping period. Females still pupped in the area, but used pieces of ice calved from the glaciers feeding into Kongsfjorden as pupping and nursing platforms (Kovacs et al. submitted). During 2007 much of the fjord remained open, but sea ice did form in one region deep in the fjord behind Lovénøyane (seven small islands) in the Northeast corner of the fjord during the spring.

### Seal Capture

Nursing bearded seal pups were captured on ice floes or in the water with a dip net as described in Lydersen et al. (1994). A total of 61 pre-weaning pups were captured over three consecutive breeding seasons (2005: n = 11, 2006: n = 16, 2007: n = 34). The status “known aged” was very conservatively assigned in this study; only pups found in the immediate proximity of their (fresh) birth sites were designated as newborns with known birth dates. For pups that were not captured and marked at their birth sites, the age at first capture was back-calculated based on body mass, co-ordination level and umbilical condition. It is relatively easy to determine that pups are less than 48 hrs old by their general lack of coordination and their fresh, often bleeding umbilicus. The umbilicus wears over the first week of life, shifting from red and very fleshy to pink and shriveled and then white and very worn. These features in combination with body mass were used to assign an age to very young pups. Pups without an umbilicus were assigned an age based on their body mass at the time of first capture ((body mass – average birth mass) ÷ average growth rate per day). The pups were also painted with an individual colour code and flipper tagged to facilitate short and long-term recognition of individuals.

At the time of each capture, the pup was weighed, and a whisker was clipped and collected. In order to maximise consistency in size and growth pattern, each whisker was collected from the same part of the muzzle, specifically the second whisker from the outside of the top line of whiskers on the left hand side. If individual seals were re-captured the new growth of the previously sampled whisker was collected, and used to estimate the whisker growth rate.

Each whisker was cleaned in chloroform and divided into 2 mm sections. Each section was analysed using an Isoprime (Micromass, UK) continuous-flow isotope-ratio mass spectrometer. Results are presented in the usual δ notation relative to Pee Dee Belemnite (PDB) and atmospheric N_2_ (Air) for C^13^ and N^15^, respectively. Replicate measurements of internal laboratory standards (acetanilide) indicate measurement errors <0.15 ‰ and <0.20 ‰ for δ^13^C and δ^15^N, respectively. Stable isotope analysis was performed at the stable isotopes Lab, by the Environmental Biology Group, Research School of Biological Sciences Australian National University (ANU).

The first 10 sections starting at the basal end of each whisker were analysed, with the basal end of the whiskers representing the most recent (post–partum) growth and the distal end the oldest sections. This was done to provide samples spanning both the pre-and post-partum growth phases of the pups growth, although only the pre-partum segments are used in this paper.

The average growth rate of pup whiskers was calculated from 20 regrowth samples collected from re-captured seals. This average rate of whisker growth was then used to estimate the point along each whisker which represented the pup's birth. This was done to exclude basal segments of whiskers, which were likely to have been produced after parturition, thereby representing pup suckling rather than maternal diet, and resulted in the between 4 and 10 pre-weaning whisker segments being used for each seal.

### Relative Breadth of Trophic Niche

The relative dietary breadth of individuals was assessed by comparing the within whisker variance [29]. As variance can be influenced by sample size, we restricted this analysis to only the four segments immediately prior to parturition (four being the number of *pre-partum* segments from the oldest whiskers). A one-way Analysis of Variance (ANOVA) was used to assess whether this varied among the years of the study.

### Inter-Annual Differences in Pre-Partum Isotopic Characteristics

We calculated the mean, standard error, maximum, minimum and range for both δ^13^C and δ^15^N of all the individual segments to characterise each whisker. These values were used to identify differences in isotopic characteristics among the three years of the study using Analysis of Similarity (ANOSIM), drawing from 10,000 random samples of the data. *Post-hoc* pair-wise comparisons were used to investigate differences between individual years, and a similarity percentage breakdown (SIMPER) analysis was used to identify which variables were most influential in accounting for the differences between the years.

### Δc^13^ And Δn^15^ Values of Potential Prey Species

A list of all known prey species of bearded seals from the Svalbard region was compiled from Hjelset et al. (1999). The δ^13^C and δ^15^N values for these and other potential prey species were obtained from Kongsfjorden with trawling (fishes) or by SCUBA diving during the open water season from late April to early September, in 1997 and 2006. Stable isotope ratios (δ^15^N and δ^13^C) of prey species were analysed at the Institute for Energy Technology (IFE), Kjeller, Norway. The samples were dried at 60–70°C to constant weight and homogenized in a mortar using a glass pestle. According to protocols of the IFE [37], lipids were removed by Soxhlet extraction for 2 h by using a solvent consisting of 93% dichloromethane (DCM) and 7% methanol, in order to reduce variability due to isotopically lighter lipid [38]. To remove traces of carbonates, the samples were acid rinsed with 2N HCl and dried at 80°C. Stable isotope ratios of the residual material were analysed on a Micromass Optima, Isotope Ratio Mass Spectrometer and expressed as per mill (‰) enrichment relative to the international standards described earlier.

In order to broadly characterise the isotopic signatures of these prey, we used a Hierarchical Cluster Analysis using the mean, variance, maximum, minimum and range of δ^13^C and δ^15^N for each species. The primary groupings derived from this analysis were allocated to ecological groups (*e.g*. benthic *vs* pelagic fish).

### Estimation of Diet Using Isotopic Mixing Models

An isotopic mixing model was used to estimate the diet of the seals using the SIAR package in R by Parnell and Jackson. This model takes the δ^13^C and δ^15^N values from a predator and fits a Bayesian model to their potential diet based on a Gaussian likelihood with a Dirichlet prior mixture on the mean [36]. Rather than include each possible prey species in the analysis, we used the mean ± SD of the δ^13^C and δ^15^N for each of the prey groups identified by the cluster analysis. Isotopic enrichment factors per trophic level of 3.4 ‰ for δ^ 15^N and 0.6 ‰ for δ^13^C [39] were applied in the models.

## Results

Ages of the 61 pups ranged from less than one day to 23 days of age, the latter being a normal weaning age for this species [5], [40]. The median age of the seals sampled was 6 days. The number of seals captured each years was 11, 16 and 34 for 2005, 2006 and 2007 respectively.

### Estimated Growth Rate of Whiskers

The 20 regrowth samples from re-captured seals had growth rates of 0.87±0.24 mm per day and 0.57±0.15 mg per day. Restricting the analysis to only those 2 mm segments grown prior to birth resulted in differing numbers of segments for each individual, ranging from 10 for those collected at birth, to only 4 for those collected at 21–23 days. As growth rates may be accelerated after cutting, these growth rates may be over-estimates of true growth rates, but this ensured that our definition of pre-partum segments was conservative, so we have assumed that no post-partum segments were included in the analysis.

### Intra-Individual Differences in Isotopes

**Figure 1:**
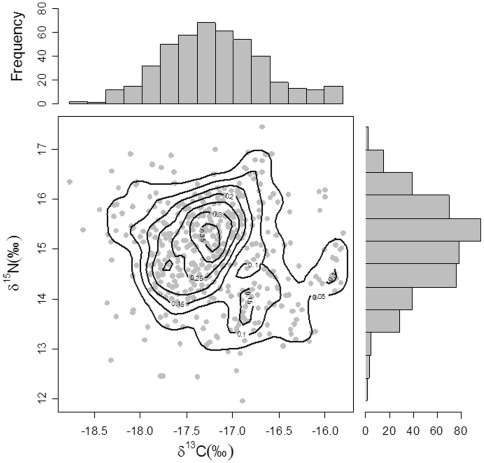
The δ^13^C and δ^15^N values for each of the 451 whisker segments from bearded seal pups, grown *pre-partum*. The marginal frequency distributions for each isotope, and in the main body of the plot, the kernel density contours are also shown.

**Figure 2:**
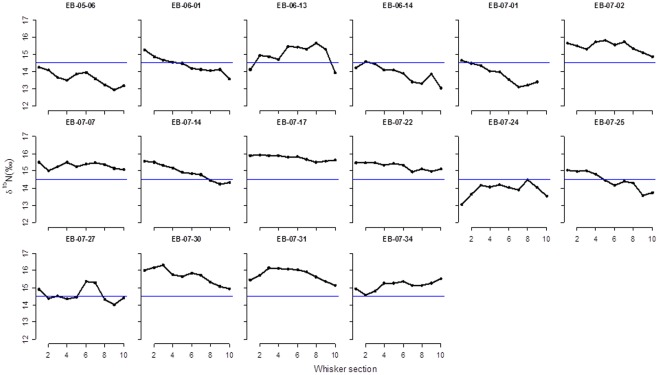
δN profiles for the 16 whiskers collected within two days of birth for bearded seals in Svalbard. The segments are numbered from the basal (most recent) to proximate (oldest) ends. The blue horizontal line represents the overall mean δ^15^N from all samples.

Overall, δ^15^N values ranged from 11.95 to 17.45 ‰ (mean±S.D. = 14.9±0.94 ‰), consistent with dietary items spanning several trophic levels (Figure 1). The isotopic values also varied considerably along the length of the individual whiskers. The full δ^15^N profiles for the 16 whiskers collected within a day of birth are presented in Figure 2. There was considerable inter- and intra-individual variation in both the basal values for δ^15^N and the nature of variation along the length of the whisker, although there was a consistent increase in δ^15^N between the distal (oldest) and basal (most recent) sections (paired t-test, t  = 2.53, d.f  = 15, p value  = 0.02).

**Figure 3:**
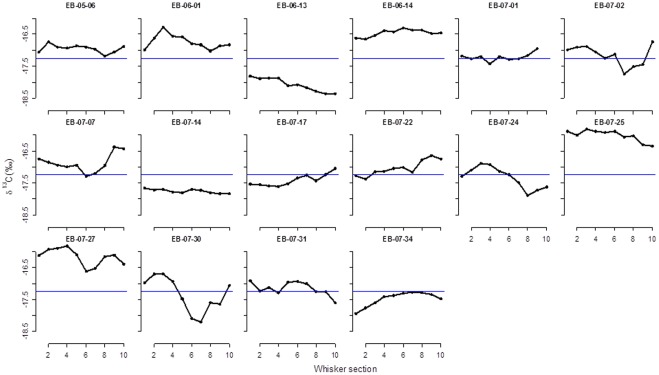
δC profiles for the 16 whiskers collected within two days of birth for bearded seals Svalbard. The segments are numbered from the basal (most recent) to the proximate (oldest). The blue horizontal line represents the overall mean δ^13^C from all samples.

There was less variability among the δ^13^C values, which ranged from −15.81 to −18.76 ‰ (mean±S.D. = −17.2±0.56 ‰, Figure 3). The full δ^13^C profile for the 16 whiskers collected within a day of birth is presented in Figure 3. There was considerable inter-individual variation in both the basal values for δ^13^C and the nature of variation along the length of the whisker, but in this case there was no significant change in δ^13^C between the distal and basal sections (paired t-test, t  = 0.35, d.f. = 15, p value  = 0.72).

Using the within whisker variance of the δ^15^N as an index of niche width for each individual, indicated that this varied by a factor of more than 3 (range = 0.6 to 2.1). The lower variance indicating a high degree of dietary specialisation, and higher variance indicating dietary generalisation.

### Inter-Annual Differences in Isotopes

The δ^15^N values differed among the three years of the study. A linear mixed effects model, comparing δ^15^N among years with individual seal as a random factor showed that 2006 (15.3±0.97) had higher values than 2005 (14.4±0.98) and 2007 (14.9±0.84) (F_2,58_ = 3.857, p = 0.02). However, δ^13^C did not vary among years (LME model, F_2, 58_ = 1.07, p = 0.35).

**Figure 4:**
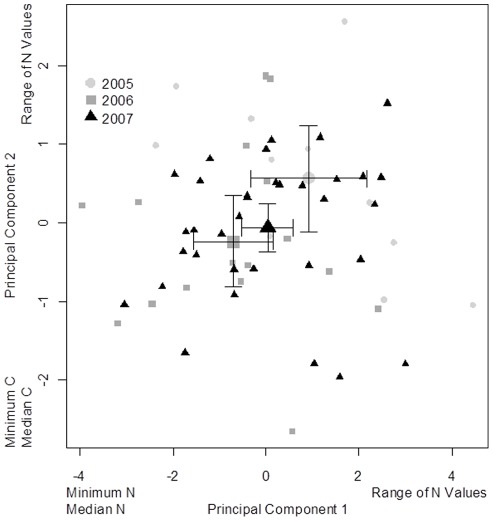
Results of the Principal Components Analysis on the whiskers of 61 bearded seal pups, using the mean, variance, maximum, minimum and range for the δ^13^C and δ^15^N for each whisker as variables in the analysis. The variables with the highest loadings are shown at each end of the axes. The mean ± SE principal component scores are also indicted for each of the three years of the study.

The ANOSIM indicated that the multivariate analysis of the combined isotopic (mean, standard error, maximum, minimum and range of δ^13^C and δ^15^N)) characteristics of the whiskers also differed among years (global R  = 0.141, p = 0.008). Examination of the pair-wise comparisons revealed that 2005 differed from both 2006 (p = 0.049) and 2007 (p = 0.017), but there was less differentiation between 2006 and 2007 (p = 0.056). This was a very similar pattern to that of δ^15^N when considered in isolation. The SIMPER analysis demonstrated that in each case the variables that contributed most to the observed differences were associated with δ^15^N, specifically the minimum, median, maximum and range of δ^15^N. The nature of the inter-annual variation is illustrated by the results of a Principal Component Analyses conducted on the same data (Figure 4). The year with the least ice in the fjords (2006) lay furthest from the year with the most ice (2005).

With respect to the trophic niche width, there was no significant differences between the within whisker variance in ^15^N among the three years (F_1,55_ = 1.66, p = 0.203).

### Isotopic Characteristics of Potential Prey Species

The mean ± S.D. isotope values for each of the potential prey species is listed in Table 1. The fish species had the highest δ^15^N values, consistent with their occupation of a higher trophic positions. There was also a wide range of δ^13^C values among the fish species, the lowest for pelagic fishes and the highest for benthic and demersal species. In contrast, the benthic gastropods and decapods had the lowest δ^15^N and highest δ^13^C values. The benthic gastropods, *Margarites* spp. had the lowest δ^13^C values of all the potential prey.

**Table 1:** Stable isotope values for 14 potential prey species of bearded seals at Svalbard. The species are group into broad taxonomic and habitat types.

Species	Taxa	Habitat	Mean δ^15^N ± S.D.	Mean δ^13^C ± S.D	n
*Boreogadus saida*	Fish	Pelagic	13.06±0.93	−20.30±0.40	25
*Buccinum finmarchianum*	Gastropod	Benthic	12.40±0.82	−18.43±0.25	3
*Buccinum glaciale*	Gastropod	Benthic	12.43±0.85	−17.90±0.30	3
*Buccinum undatum*	Gastropod	Benthic	11.80±0.58	−16.95±0.30	6
*Clupea harengus*	Fish	Pelagic	13.84±1.67	−20.80±0.36	9
*Gadus morhua*	Fish	Benthopelagic	13.98±0.82	−19.39±0.44	17
*Hippoglossoides platessoides*	Fish	Demersal	14.27±0.76	−19.08±0.49	11
*Hyas araneus*	Decapod	Benthic	11.32±0.83	−18.63±1.00	9
*Leptoclinus maculatus*	Fish	Demersal	13.38±0.80	−18.45±0.58	19
*Lumpenus lumpretaeformis*	Fish	Demersal	14.13±0.37	−17.72±0.29	9
*Lycodes* sp.	Fish	Demersal	14.94±0.71	−18.34±0.64	5
*Margarites groenlandicus*	Gastropod	Benthic	8.80±0.00	−18.30±0.14	2
*Margarites helicinus*	Gastropod	Benthic	8.85±0.35	−17.05±0.35	2
*Reinhardtius hippoglossoides*	Fish	Benthopelagic	13.99±0.80	−19.74±0.52	9

**Figure 5:**
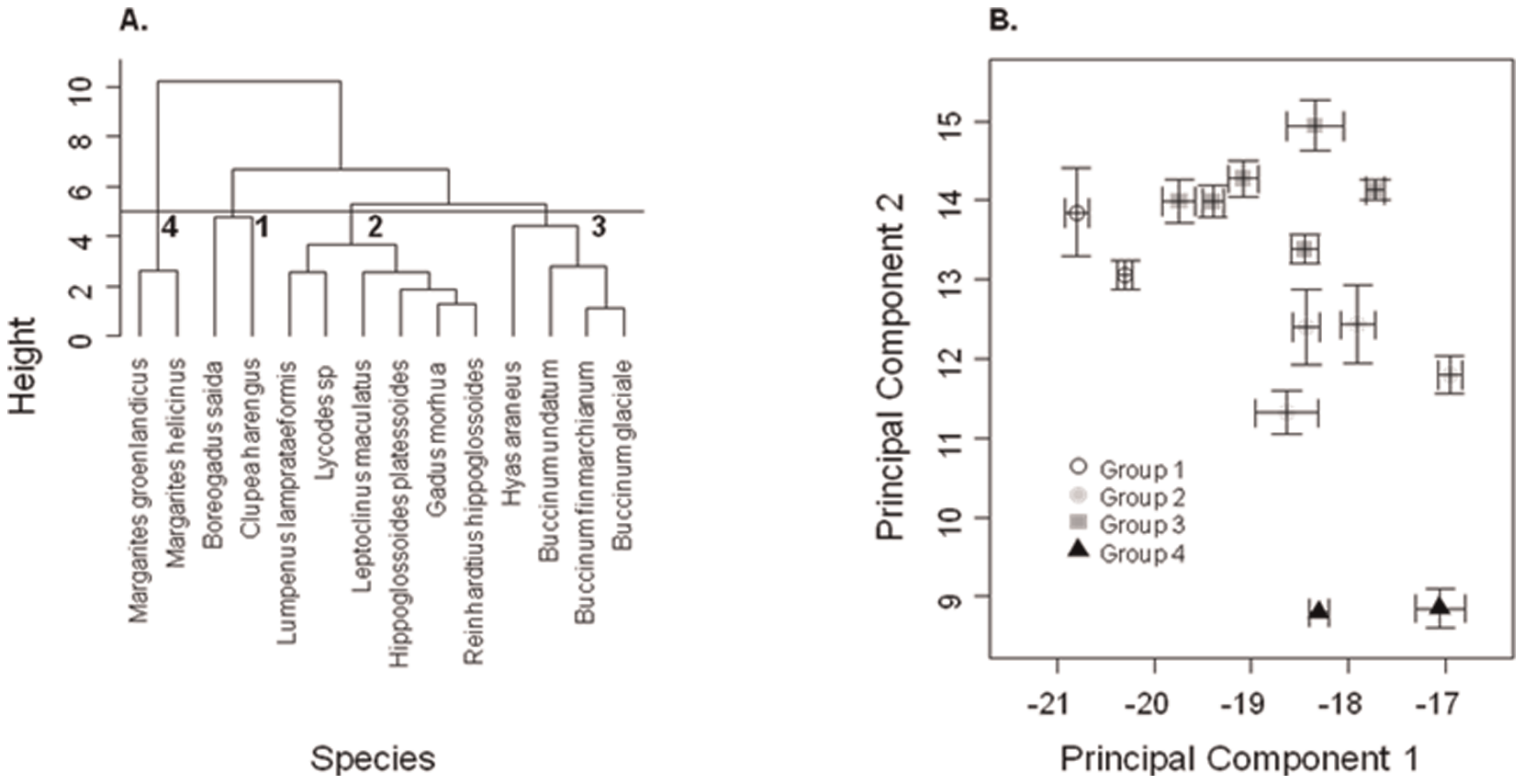
Results of the Cluster Analysis on the 14 potential prey species showing (a) the dendrogram indicating the 4 principal groups, and (b) the mean (±95% confidence limits) δ ^13^C and δ ^15^N values for each of the prey species. The different colours indicate prey groups assigned by the cluster analysis.

These general patterns of isotopic signatures were reflected in the cluster analysis, with four clear groups of prey species (Figure 5a). The groups were (1) two pelagic fish species, polar cod (*Boreogadus saida*) and herring (*Clupea harengus)* (2) Six species of demersal/benthic fish, (3) four species of benthic gastropods and decapods and (4) two species of the benthic gastropod from the genus *Margarites*.

### Diet of Gestating Bearded Seals

**Figure 6:**
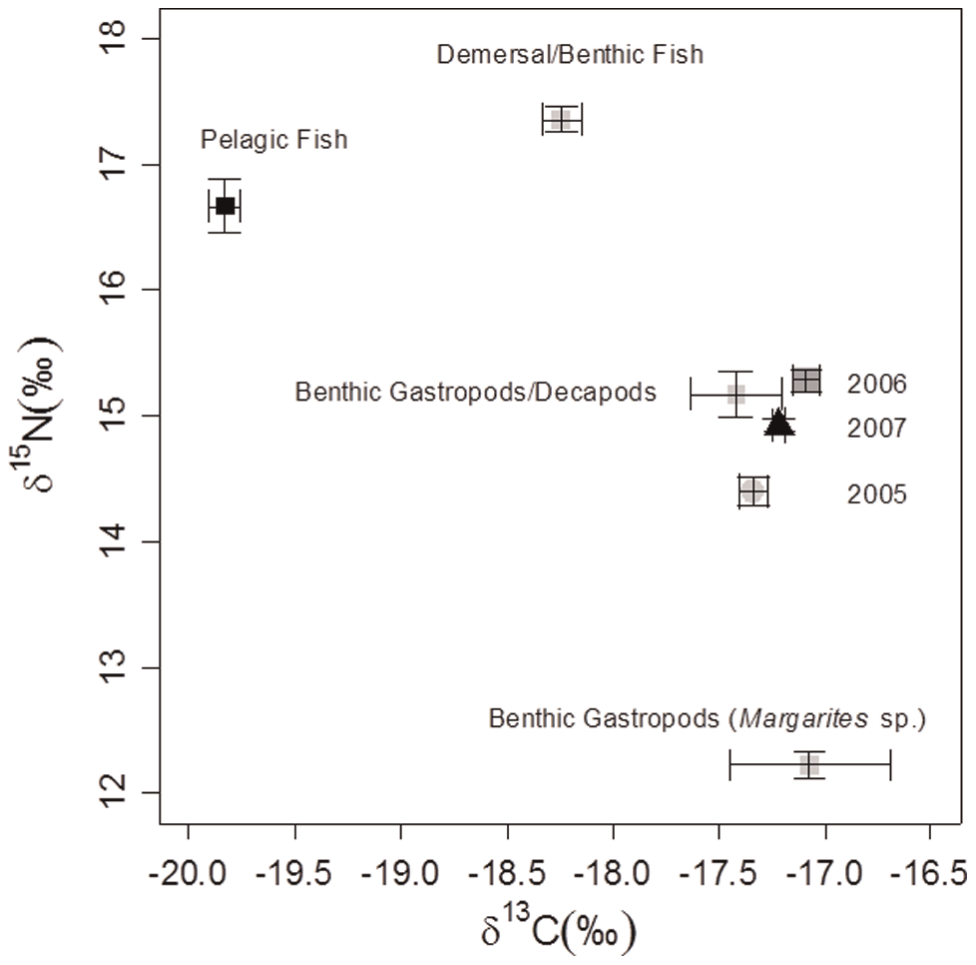
Plots of the mean δ^15^N and δ^13^C values (mean±S.E.) for each whisker segment in each of the three years of the study (filled black circles and associate error bars). The open squares are the mean δ^15^N and δ^13^C values of the potential prey species using the groupings established in Figure 6. Note that the values for the seals have been adjusted by 3.4‰ to allow for the trophic enrichment.

**Figure 7:**
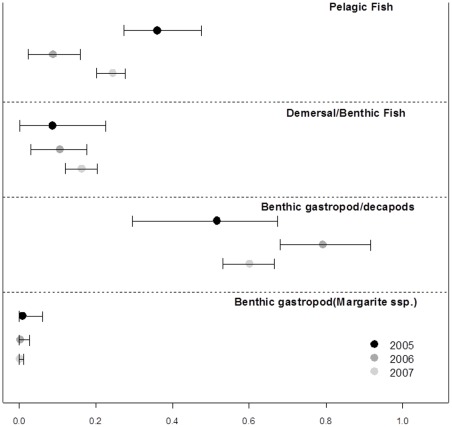
Estimated diet composition of bearded seals in the three years of the study. The modal value and the 95 confidence limits resulting from the SIAR R package are indicated. The analysis was based on four principal prey groups, contrasting each of three years within each group.

When we compare the mean isotopic values for the seals in each year to those of the prey species (Figure 6) the bearded seal data lay closest to the benthic gastropod and decapod group, which itself was mid-way between the fish and *Margarites* groups. However, as the values from the whiskers could be the result of a range of possible mixes of diet species, this simple comparison is of limited value. Isotopic mixing models are designed to estimate the most likely combination of prey types that will be responsible for a particular set of predator isotope values. In our case, the mixing models were run using the four groups of prey identified by the cluster analysis. The models indicated that the seals fed predominantly on benthic gastropods and decapods, which constituted 50–80% of their diet (Figure 7). The benthic gastropods (*Margarites* spp.) were the least common, and made up <10% of the diet in all years. The demersal and benthic fish groups varied little among the years of the study representing between 10–20% of the overall diet. The pelagic fish group demonstrated the greatest inter-annul variation. This group represented 30–50% of the diet in 2005, similar to the proportion of benthic gastropods and decapods in the diet that year. But, pelagic fish were least prevalent in 2006 (<20%), and this was also the year with the greatest proportion of benthic gastropods and decapods (>70%). 2007 was intermediate between these years with respect to both the pelagic fish and benthic gastropod components of the diet.

## Discussion

This study has demonstrated that stable isotopes in the whiskers grown by bearded seal pups *in utero* can be used to infer the dietary trends of their mothers. Further, the diets of the pregnant females varied over the course of the three year study. In the year with the most extensive fast ice the diet consisted of more pelagic fish species and fewer benthic invertebrates, while the opposite was true in the years when the fjords were relatively ice free. Therefore, the diet of pagophilic (ice–loving) species such as bearded seals can be influenced by changes in physical characteristics of their environment. There may be long-term changes in diet if ice conditions continue to deteriorate, which is predicted for the coming decades, with the possibility of an ice-free Arctic summer by 2035 [41].

### Growth Rate of Pup Whiskers

Using regrowth of whiskers cut on the initial capture we estimated growth rates of post-partum whiskers to be 0.87 mm and 0.57 mg per day. Whisker growth rates of free-ranging seals have rarely been estimated, but the available information indicates considerable variability among species. Captive adult harbour seals (*Phoca vitulina*) had mean whisker growth rates of 0.08 mm.day^−1^, although there is considerable intra-annual variability with growth rates as high as 0.60 mm.day^−1^ recorded in summer [42]. Captive grey seals (*Halichoerus grypus*) had mean whisker growth rates of 0.24 mm.day^−1^, but there were also periods of accelerated growth, although no specific data were provided [43]. Whiskers of newly weaned southern elephant seals (*Mirounga leonina*) grow at an average rate of 0.22 mm.day^−1^ during the first few months at sea (A. Walters unpublished data). In all cases, the mean growth rates were lower than our estimate, although the periods of higher growth may be more congruent with those of the bearded seals pups. However, none of these earlier studies are based on pre-*partum* or nursing seal pups, and these periods are times of rapid somatic growth that are likely also reflected in the growth of the whiskers. Methodological differences may also account for the observed differences, particularly in our study which used regrowth of cut whiskers which may have resulted in accelerated growth.

### Intra-Individual Differences in Isotopes

One of the great strengths of stable isotopes as a source of dietary information lies in their sequential incorporation in growing tissues, providing a history of prey consumption over a period of days or even months [44], [45]. Pinniped whiskers are increasingly being used for this task [46], although full interpretation of such time series requires a sound understanding of whisker growth dynamics [47]. Using the whiskers of newborn pups overcomes some problems as the history of each whisker is entirely *in utero*, unlike older animals which will have experienced moulting and whisker wear, making the history of individual whiskers uncertain.

Bearded seals are commonly described as generalist feeders, although they predominantly feed on benthic prey [11], [48]. The within whisker variance of the δ^15^N (an index of niche width for each individual) indicated considerable variability in maternal foraging patterns. Flexibility in diet (indicated here by higher variance) is often cited as an indication that a species will be less sensitive to environmental change, particularly compared to dietary specialists [11], [49]. Earlier, conventional diet studies for this species were not able to address this important issue [10]. Our results confirm that bearded seals are dietary generalists at a population/ species level, but that some individuals appear to be dietary specialists.

Among the newborn pups (which had the longest sequence of pre-partum segments) δ^15^N varied by <0.5‰ in some individuals and by 2.0 ‰ in others. The seals with the least variation were specialising on high trophic level prey, while those with the greatest variation contained a mix of higher and lower trophic level prey. This may reflect the foraging habitats chosen by the mothers, as individuals remaining further offshore in deeper waters may not have access to benthic prey to the same degree as mothers feeding closer to shore. There were however, an increase in the δ^15^N values as parturition approaches, and the reasons for this are unclear. It could represent a genuine shift in diet to larger more energy dense (lipid rich) prey in the lead up to parturition, or it may simply be due to females fasting for several days, and the pups nitrogen signature therefore reflecting that of the mother rather than her prey [50].

A question that arises is “does the degree of dietary specialisation reported here indicate a diet or habitat preference that is immutable, or does it simply reflect local conditions at a particular time or place”? High levels of individual specialisation in foraging traits have been reported in several pinniped species. For example, individual southern elephant seals exhibit diving behaviours different from other con-specifics foraging in the same water masses, and individuals have high levels of foraging site fidelity, returning the same regions to forage year after year [51], [52]. The degree to which individual seals can modify their foraging patterns is the true measure of how susceptible a species will be to environmental change, rather than a single measure of population dietary diversity. Our data, suggest that individual bearded seals are capable of responding to environmental change, as there were significant difference in diet among the years, which corresponded to differing ice conditions.

### Diet of Late-Gestation Bearded Seals

The diet of bearded seals is reasonably well described across their range [53], [54] and it has been studied in Svalbard [17]. But, none of these earlier studies have examined the crucial late gestation period, when females need to acquire energy to fuel the growth of the pup as well as accumulating fat stores to help with the upcoming energetic costs of lactation [5], [55]. While stable isotope analysis can provide powerful insights into temporal and spatial differences in trophic relationships between predators and their prey, using the data to infer the actual diet can be more problematic. There are two broad approaches to inferring diet from δ^15^N and δ^13^C. The first is relatively qualitative, and compares the isotope values from the predator to those of potential prey species. For the Svalbard region we had δ^15^N and δ^13^C values for most of the common bearded seal prey items [17], and a qualitative comparison indicated that the mean bearded seal isotopic values clustered closest to the values for benthic gastropods and decapods. This indicates that these prey items are likely to be very important for bearded seals, but this interpretation fails to take into account the alternative explanation, *e.g*. that these stable isotope values are the result of a mixed diet consisting of fish and *Margarites spp*. (as the seal values lie almost mid-way between these two prey groups). Mixing models have been developed to help determine which of all the possible diet combinations are the most likely to result in the observed isotope ratios [56], [57], [58]. The uncertainty associated with the resulting models can be considerable. It is a function of the variability in the isotopic values of the prey, the variability in the isotope value of the predator and the range of potential prey types as well as the degree of fractionation between trophic levels. However, modern models use Bayesian approaches that estimate probability distributions of prey contributions to potential diet mixes while explicitly accounting for uncertainty associated with multiple prey [57]. We have attempted to further minimize uncertainty through reducing the number of prey types by aggregating the individual prey species into four groups, and expressing the diet in terms of these groups rather than for individual species.

The results of the mixing models confirmed that benthic gastropods and decapods constituted the largest proportion of the diet. The *Margarites* spp. gastropods were not particularly important, while fish, and in particular the pelagic fish, constituted up to 40% of the diet. This result did not emerge from the qualitative comparison of predator and prey isotopes, as the pelagic fish were quite distinct from the seals. Nonetheless, our final estimate of diet is entirely consistent with previously reported diet (albeit from a different time of year) for bearded seals in Svalbard [17]. Caution is required when comparing across such different techniques however, and the only valid comparison would be from isotopic analysis of tissues and prey collected throughout a whole year.

### Inter-Annual Differences in Diet

We used pups as a vehicle for studying adult female diet, due to the difficulty of live-catching a reasonable number of adult females during the later winter and early spring. One disadvantage of this approach is that we were unable to identify individual mothers, and in particular would not know if the same individuals were sampled in subsequent years. Such data on inter-annual diet of individuals would help to determine whether or not individual seals were switching their diet as a consequence of the contrasting ice conditions in the fjord. However, more pups were born in Spitsbergen fjords in the years of reduced sea ice, which was due to females seeking floating platforms on which to pup and nurse. In both 2006 and 2007, the bearded seals mainly used floating glacier ice rather than sea-ice for these activities, as this was the only available to them. This ice was mainly accessible close to the glacier fronts in the inner parts of the fjords, which had the effect of bringing greater number of seals into the fjords. In a “normal” sea ice year such as 2005 these seals would have pupped further offshore on ice floes close to the fast ice edge. In this year, when the seals were further offshore and in deeper water, their diet was dominated by pelagic fish. The lack of sea ice in 2006 and 2007 concentrated pregnant female bearded seals in glacier ice calving regions, resulting in the higher than normal pup densities. This also inevitably resulted in the seals spending more time in shallow very close inshore waters, which is reflected in the predominance of benthic prey in those years.

The lack of difference in δ^13^C among the years of the study is not surprising, even though carbon can be a good marker for onshore *vs* offshore habitats. In our study site, the geographic difference between high and low ice years is on the order of only 10 km. While this is sufficient to expose the gestating females to different water depths (and therefore potentially different prey), this may not be reflected in carbon which is similar across the shelf due to advection of Transformed Atlantic Water into the fjords. Only by integrating the predator carbon and nitrogen data with those of known prey species in mixing models were we able to elucidate the changing pattern of diet in the seal during the late winter and spring.

The mothers' primary requirements for breeding habitat determine where they feed during the breeding season, but this has consequences for the types of prey consumed during late gestation. The observation that in 2005, during the “normal ice” year with regard to fast ice in the fjords, females consumed the greatest proportion of pelagic fish and the lowest proportion of benthic invertebrates. Long-term trends of sea ice decline, earlier ice melt, and increased water temperatures in the Arctic [59] are likely to have ecosystem-wide effects. For bearded seals in Svalbard, this will mean inhabiting and pupping on glacier ice near the heads of fjords, with an associated increase in benthic gastropods and decapods in their diet. While the biological implications of this change are uncertain, this does illustrate that the way that polar species in general will respond to long-term changes in sea-ice conditions will be dependent on a range of species-specific physical and biological factors, making it difficult to predict the nature of ecosystem effects with any degree of certainty.
